# Breakfast Habits in Patients Using Levothyroxine: Patient Experiences and Preferences

**DOI:** 10.1210/jendso/bvae180

**Published:** 2024-10-21

**Authors:** Jeresa I A Willems, Daan J L van Twist, Inge H Y Luu, Rutgert Bianchi, Robin P Peeters, Roderick F A Tummers-de Lind van Wijngaarden

**Affiliations:** Department of Internal Medicine, Zuyd Thyroid Center, Zuyderland Medical Center, 6162 BG Sittard-Geleen, the Netherlands; Department of Internal Medicine, Zuyd Thyroid Center, Zuyderland Medical Center, 6162 BG Sittard-Geleen, the Netherlands; Department of Internal Medicine, Zuyd Thyroid Center, Zuyderland Medical Center, 6162 BG Sittard-Geleen, the Netherlands; Department of Internal Medicine, Zuyd Thyroid Center, Zuyderland Medical Center, 6162 BG Sittard-Geleen, the Netherlands; Academic Center for Thyroid Diseases, Department of Internal Medicine, Erasmus Medical Center, 3015 GD Rotterdam, the Netherlands; Department of Internal Medicine, Zuyd Thyroid Center, Zuyderland Medical Center, 6162 BG Sittard-Geleen, the Netherlands

**Keywords:** levothyroxine, fasting ingestion, breakfast, hypothyroidism

## Abstract

**Background:**

Levothyroxine (LT4) is recommended to be ingested in a fasting state, 30-60 minutes before breakfast to avoid interactions with food and drugs. In clinical practice, we noticed that this instruction may be inconvenient for patients. Therefore, we aimed to evaluate patient experiences and preferences concerning the recommended fasting administration of LT4.

**Methods:**

Patients using LT4 were invited to complete a questionnaire. Regression analyses were performed to identify patient characteristics associated with taking LT4 close to or together with food and/or interfering drugs, feeling burdened with postponing breakfast, and preferring nonfasting LT4 ingestion.

**Results:**

Of 463 invited patients, 410 completed the questionnaire (88.6%). Of these, 76.8% was female and median age was 57 years (interquartile range: 43-67). Nearly all patients (97.3%) reported to have received instruction on fasting LT4 ingestion, but only 30% adhered to this. Nonfasting LT4 intake was associated with use of co-medication (odds ratio [OR], 2.82; 95% CI, 1.77-4.47), treatment duration >1 year (OR, 1.76; 95% CI, 1.02-3.04), and male sex (OR, 1.67; 95% CI, 1.03-2.70). Approximately half of the patients reported being burdened with postponing breakfast and the majority (60.5%) expressed their preference for nonfasting LT4 ingestion. Interestingly, 25% omitted breakfast and 13.4% forgot their medication because of the fasting requirement. Furthermore, the majority (68.2%) of patients that used interfering drugs stated not to be instructed to separate these drugs from LT4.

**Conclusion:**

This study highlights the burden associated with fasting LT4 ingestion, leading to nonadherence, irregular LT4 intake, and omitting breakfast. Given the clear preferences towards nonfasting LT4 ingestion, further research into alternative nonfasting administration methods is warranted.

Levothyroxine (LT4) is recommended to be ingested in a fasting state and separated from interfering drugs [1]. Food intake and several medications (eg, antacids, calcium supplements) can elevate gastric pH, thereby impairing the disintegration and dissolution of LT4 in the stomach before its intestinal absorption. Furthermore, specific foods and drugs bind to LT4 (as seen with coffee, milk, fiber, and soy products), form insoluble complexes with LT4 (as seen with calcium, iron, aluminum, and magnesium supplements), or impair its absorption by competing with its transporters (as seen with fruit juices) [2, 3]. Hence, current guidelines recommend taking LT4 30 to 60 minutes before breakfast and to separate it from interfering drugs for at least 4 hours to optimize absorption [1].

Among patients with hypothyroidism, up to 43% of patients do not reach their target thyroid-stimulating hormone (TSH) level, despite LT4 treatment [4-7]. Together with nonadherence, food and drug interactions may play a pivotal role in these disappointing treatment results. In clinical practice, we notice that the requirement to postpone breakfast for LT4 ingestion is inconvenient for many patients. On the one hand, this may influence LT4 absorption in case of ingestion together with food or medication. On the other hand, however, these inconveniences may also affect adherence to LT4 ingestion itself. Yet, data on how these recommendations impact patients' daily routines are limited. Therefore, we aimed to evaluate patient experiences and preferences concerning the recommended fasting administration of LT4 in relation to breakfast habits, using patient-reported experience measures.

## Materials and Methods

Physicians were instructed to invite all patients who used LT4 and who visited the internal medicine outpatient clinic of Zuyderland Medical Center, The Netherlands, during a 6-month study period, to complete a questionnaire. All patients aged ≥18 years and who used LT4, irrespective of the cause of hypothyroidism, were eligible for participation in this study. Patients who were unable to comprehend and complete the questionnaire were excluded. All patients provided written informed consent. This study was approved by the institutional ethical review board (METCZ20230014).

### Questionnaire

Eligible patients were requested to complete a digital patient-reported experience measure questionnaire (Supplemental File) [8] with questions on (1) demographic characteristics, (2) quality of life (QoL), (3) course of thyroid disease and subsequent LT4 treatment, (4) experiences and preferences concerning the fasting intake of LT4, (5) instructions received from their physician or pharmacist to separate LT4 from food and interfering drugs, and (6) adherence to these instructions. Patients who reported inconveniences with postponing breakfast were asked to rate the level of burden on a 10-point Likert scale, where 0 indicated no burden and 10 signified severe burden. Health-related QoL was evaluated by using the visual analogue scale of the EQ-5D instrument (validated version in Dutch language) [9], where 0 represented the worst health the patient could imagine and 100 indicated the best imaginable health. The impact of thyroid disease on QoL was assessed with a thyroid-specific QoL question derived from the validated Dutch version of the ThyPRO-39 questionnaire [10]. Highly educated was defined as completion of university or higher professional education, whereas completion of secondary vocational education, secondary school, or primary school was considered as low educated [11]. Shift work was considered as working irregular hours and rotating through different shifts. Parenting young children was considered as living with children aged <12 years. All data were collected anonymously and incomplete surveys were excluded from analysis.

### Laboratory Evaluation

We used the most recent serum TSH (reference range, 0.27-4.20 mIU/L) and free thyroxine (FT4, reference range, 11.0-22.0 pmol/L) levels, measured as part of patients' regular outpatient follow-up up to 6 months before or after completion of the questionnaire. TSH and FT4 were measured with a Cobas Pro analyzer (Roche, Basel, Switzerland) using sandwich immunoassays.

### Statistical Analysis

Categorical variables were expressed as frequencies and percentages. Continuous data were all nonparametric and reported as medians with interquartile range (IQR). Comparisons between groups were calculated using the chi-square test or Fisher's exact test for categorical variables and Mann-Whitney *U* test for continuous variables, as appropriate. Logistic regression was used to identify patient characteristics associated with nonfasting LT4 ingestion, improper LT4 ingestion close to or together with food and/or interfering drugs, being burdened with postponing breakfast, and preference for nonfasting LT4 ingestion. For each of these dependent variables, age, use of co-medication, gender, educational level, parenting young children, shift work, and treatment duration were used as covariates. All covariates were assessed as categorical variables, except age, which was analyzed as a continuous variable. All covariates were included in the multivariate logistic regression model, regardless of their significance in the univariate logistic regression model because of their presumed plausibility in influencing potential risk factors. Odds ratios (ORs) with 95% CIs were calculated. *P* values < .05 were considered statistically significant. Statistical analysis was conducted using SPSS software (version 28.0, IBM, Corp., Armonk, NY, USA). Figures were created using Figlinq (https://figlinq.com) and BioRender (https://www.biorender.com) platforms.

## Results

Between April 14, 2023, and October 7, 2023, a total of 463 patients treated with LT4 were invited to participate in this study, of whom 410 (88.6%) completed the questionnaire. Baseline characteristics of the study cohort are summarized in Table 1. The majority of patients were female (76.8%) and median age was 57 years (IQR: 43-67). Hashimoto's disease was the most common reason for LT4 treatment (55.6%). The median daily LT4 dose was 100 mcg (IQR: 75-125) and 81.2% of patients had been under treatment for more than a year. LT4 was primarily administered in tablet form (93.4%), whereas only a minority used soft gel capsules (6.6%), and none used liquid LT4. The median TSH level was 1.35 mIU/L (IQR: 0.48-3.17) and 66.2% of patients had TSH levels within the reference range. TSH levels were measured within a median of 8 days (IQR: 4-14) before or after completion of the questionnaire.

**Table 1.** Baseline characteristics of the study population

N	410
**Demographics**	
Age (y)	57 [43-67]
Female, n (%)	315 (76.8)
Low educational level, n (%)	265 (64.6)
Shift work, n (%)	32 (7.8)
Parenting young children, n (%)	129 (31.5)
**Etiology of hypothyroidism**	
Hashimoto's disease, n (%)	228 (55.6)
Post RAI, n (%)	46 (11.2)
Block and replace treatment for Graves' disease, n (%)	42 (10.2)
Postsurgery, n (%)	36 (8.8)
Thyroid cancer, n (%)	24 (5.9)
Drug-induced, n (%)*^a^*	18 (4.4)
Other, n (%)*^b^*	16 (3.9)
**LT4 treatment**	
Dose (mcg)	100 [75-125]
Treatment in secondary care, n (%)	330 (80.5)
Duration of treatment	
≤1 y, n (%)	77 (18.8)
>1 and ≤ 10 y, n (%)	168 (41)
>10 y, n (%)	165 (40.2)
LT4 formulation	
Tablet, n (%)	383 (93.4)
Soft gel capsule, n (%)	27 (6.6)
Liquid, n (%)	0 (0)
**Laboratory analysis**	
TSH (mIU/L)	1.35 [.48-3.17]
FT4 (pmol/L)	18.4 [15.10-21.10]
TSH within reference range, n (%)*^c^*	251 (66.2)
TSH below reference range, n (%)*^c^*	68 (17.9)
TSH above reference range, n (%)*^c^*	60 (15.8)
Time between blood test and completion questionnaire (days)	8 [4-14]

Values are presented as n (%) or median [interquartile range]. Definitions of educational level, shift work, and parenting young children are provided in the Methods section.

Abbreviations: FT4, free thyroxine; LT4, levothyroxine; RAI, radioactive iodine; TSH, thyroid-stimulating hormone.

^*a*^Amiodarone- (n = 5) or immunotherapy-induced hypothyroidism (n = 13).

^*b*^Consisting of hypopituitarism (n = 11), suppressive therapy for euthyroid multinodular goiter (n = 2), postpartum thyroiditis (n = 1), subfertility due to subclinical hypothyroidism (n = 1), and unknown cause (n = 1).

^*c*^Reference interval of the laboratory of Zuyderland Medical Center was used (TSH: 0.27-4.20 mIU/L) and 31 patients without available TSH measurement within 6 months before or after completion of the questionnaire were excluded from the denominator.

### Patient-reported Experiences and Preferences

**Figure 1.**
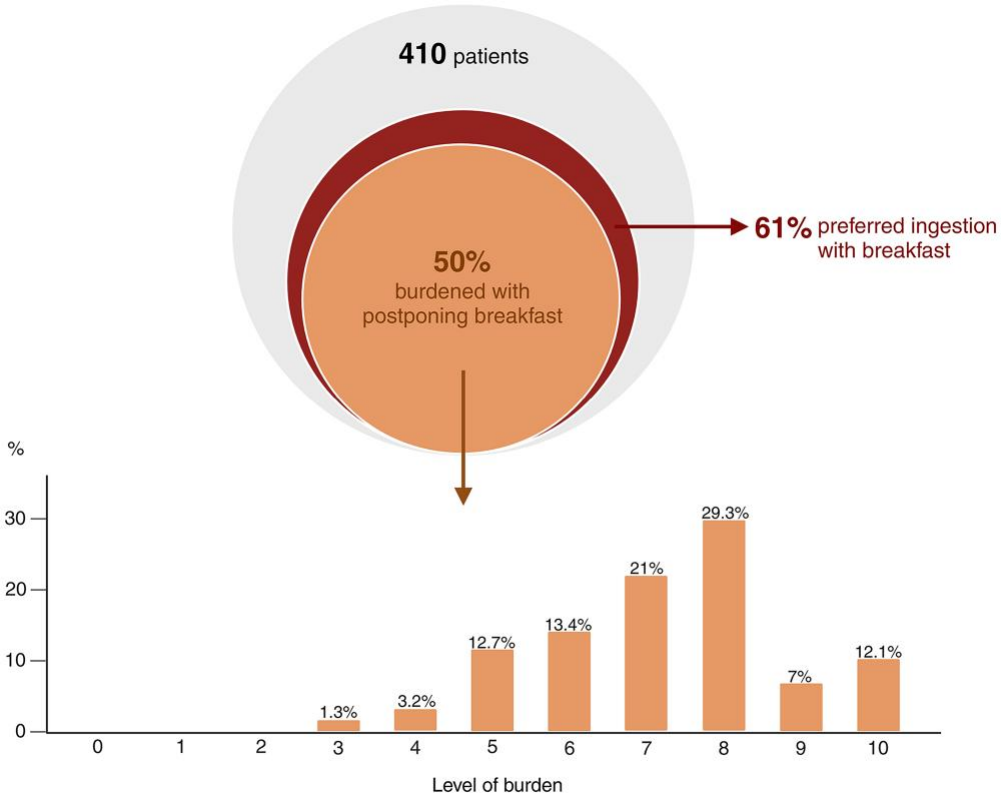
Patient-reported burden and preference concerning LT4 ingestion. The level of burden felt with postponing breakfast was reported on a 10-point Likert scale (0, no burden; 10, severe burden).

A total of 97.3% (n = 399) reported taking LT4 in the morning with 68.9% (275 of 399 patients) adhering to the 30-60 minutes prebreakfast recommendation (Table 2). Eleven patients (2.7%) reported nonmorning ingestion yet ensured to separate LT4 from the adjacent meal. Although 89.5% of patients reported to have received instruction to take LT4 in a fasting state, 60.5% expressed their preference for nonfasting LT4 ingestion because of the waiting time until breakfast (Fig. 1). Nearly half of the patients (49.5%) felt burdened with the need to postpone breakfast, rating the burden with a median score of 7 (IQR: 6-8) on a Likert scale from 0 (no burden) to 10 (severe burden; Fig. 1). Notably, 24.9% of patients chose to omit their breakfast because of the fasting requirement and 13.4% forgot to administer LT4 because of this instruction. Finally, 62% of patients stated that their thyroid disease, irrespective of LT4 treatment, negatively affected their QoL.

**Table 2.** Patient-reported habits, experiences, and preferences concerning fasting LT4 ingestion

N	410
**Patient-reported timing of LT4 ingestion**	
30-60 min before breakfast, n (%)	275 (67.1)
< 30 min before breakfast, n (%)	92 (22.4)
Together with breakfast, n (%)	32 (7.8)
Before bedtime, at night, or alternating ingestion methods, n (%)	11 (2.7)
**Patient-reported experiences and preferences**	
Received instruction on fasting ingestion, n (%)	367 (89.5)
Considered fasting ingestion as postponing breakfast, n (%)	248 (60.5)
Preferred ingestion together with breakfast, n (%)	248 (60.5)
Burdened with postponing breakfast, n (%)	203 (49.5)
Level of burden*^a^*	7 [6-8]
**Patient-reported adverse effects because of fasting LT4 ingestion**	
Omitting breakfast, n (%)	102 (24.9)
Forgetting LT4, n (%)	55 (13.4)
**Patient-reported use of co-medication**	138 (33.7)
Potentially interfering drugs, n (%)	85 (61.6)
Received instruction on separating interfering drugs, n (%)	27 (31.8)
Timing of ingestion	
Together with LT4, n (%)	57 (67.1)
≤ 1 h before/after LT4, n (%)	17 (20.0)
≥ 4 h before/after LT4, n (%)	11 (12.9)
**Patient-reported QoL**	
Health score*^b^*	70 [60-80]
Thyroid disease negatively affected QoL, n (%)*^c^*	254 (62.0)

Values are presented as n (%) or median [interquartile range]. The definition of interfering drugs is provided in the Methods section.

Abbreviations: LT4, levothyroxine; QoL, quality of life.

^*a*^Reported on a 10-point Likert scale (0, no burden; 10, severe burden).

^*b*^Reported on a 0 to 100 visual analogue scale; question (“How good is your health today?”) derived from the EQ-5D instrument.

^*c*^Thyroid-specific QoL question (“Did your thyroid disease negatively affected your QoL in the previous month?”) derived from the ThyPRO-39 questionnaire.

### Patient-reported Use of co-medication

Among the study population, 33.7% (n = 138) reported use of other medication alongside LT4. Of those, 61.6% (85 of 138 patients) used drugs and/or supplements known to interfere with LT4 absorption including calcium-, iron-, and magnesium-containing supplements, as well as other vitamins, potassium and phosphate binders, proton pump inhibitors, and H_2_-receptor antagonists. Of the 85 patients who used interfering medication, only 27 (31.8%) indicated having received instruction to maintain a 4-hour interval between the interfering drug and LT4, with 40.7% (11 of the 27 patients) adhering to this instruction.

### Risk Factors

**Figure 2.**
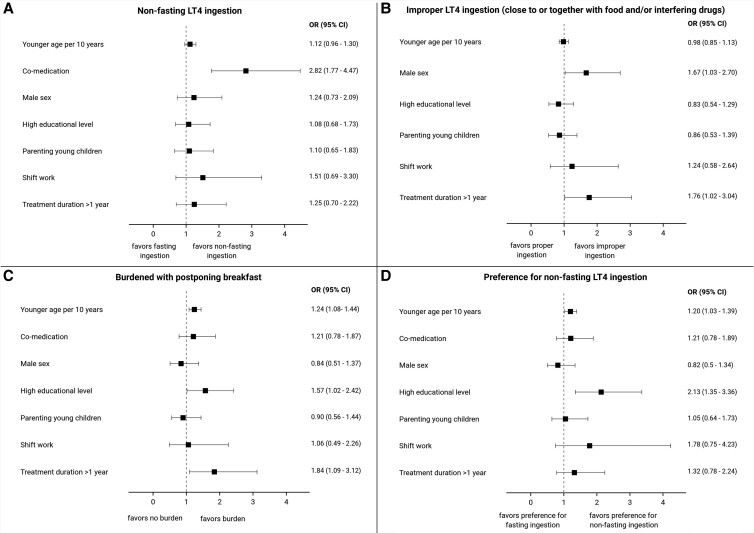
Forest plots for associations between several patient characteristics and taking LT4 in a nonfasting state (A), taking LT4 improperly close to or together with food and/or interfering drugs (B), feeling burdened with postponing breakfast (C), and preferring nonfasting LT4 ingestion (D). Definitions of educational level, shift work, and parenting young children are provided in the Methods section.

Abbreviations: LT4, levothyroxine; OR, odds ratio.

In both univariate and multivariate logistic regression analysis, there was a statistically significant association between nonfasting LT4 ingestion and use of co-medication (OR, 2.82; 95% CI, 1.77-4.47; *P* < .001; Fig. 2 and Supplemental File [8]). Improper LT4 intake close to or together with food and/or interfering drugs was associated with a treatment duration >1 year (OR, 1.76; 95% CI, 1.02-3.04; *P* = .04) and male sex (OR, 1.67; 95% CI, 1.03-2.70; *P* = .04). Feeling burdened with postponing breakfast and preferring nonfasting LT4 ingestion were associated with a higher educational level (OR, 1.57; 95% CI, 1.02-2.42; *P* = .04 and OR, 2.13; 95% CI, 1.35-3.36; *P* = .001, for feeling burdened with postponing breakfast and preferring nonfasting LT4 ingestion, respectively) and younger age (OR, 1.24; 95% CI, 1.08-1.44; *P* = .003 and OR, 1.20; 95% CI, 1.03-1.39; *P* = .02, for feeling burdened with postponing breakfast and preferring nonfasting LT4 ingestion, respectively). In addition, patients treated with LT4 for >1 year were more likely to feel burdened with postponing breakfast (OR, 1.84; 95% CI, 1.09-3.12; *P* = .02). We found no statistically significant association between feeling burdened with postponing breakfast and QoL (OR, 1.12; 95% CI, .98-1.27; *P* = NS) or thyroid-associated QoL (OR, 1.43; 95% CI, .91-2.26; *P* = NS). Surprisingly, patients who reported to ingest LT4 in a nonfasting state were more likely to have a TSH value within the reference range (OR, 1.59; 95% CI, .97-2.58; *P* = .06). TSH outside the reference range was not associated with LT4 co-ingestion with interfering drugs (OR, .63; 95% CI, .35-1.15; *P* = NS), lower QoL (OR, .98 per 10 points on EQ-5D; 95% CI, .86-1.13; *P* = 0.NS) or thyroid-associated QoL (OR, 1.19; 95% CI, .76-1.86; *P* = NS).

## Discussion

In this study, we evaluated patient experiences and preferences concerning fasting LT4 ingestion in 410 patients. Although almost all patients (89.5%) reported having received instruction to take LT4 in a fasting state, approximately 1 of 3 patients (30.2%) did not adhere to this instruction. Nearly half of the patients felt burdened with the waiting time until breakfast and 60.5% would prefer nonfasting LT4 ingestion. Notably, 24.9% omitted breakfast and 13.4% forgot their medication, both because of the fasting requirement.

Despite clear instructions on the importance of fasting LT4 intake, >30% of the patients in this study chose to ingest LT4 close to or together with breakfast. Previous studies in similar cohorts showed even higher nonfasting ingestion rates ranging from 41.5% to 85.4% [7, 12]. Specific reasons for nonadherence to this fasting recommendation are not fully elucidated yet, but we observed higher rates of nonfasting LT4 intake among patients treated for a longer period, among male patients, and among patients using co-medication. Among the latter group, only a minority of patients that used interfering medication (31.8%) stated having received instruction to separate the interfering drug from LT4. Of those, 60% did not maintain the recommended 4-hour separation between both drugs, a remarkable finding, which is in line with previous studies [7]. These findings not only demonstrate the limited adherence to fasting ingestion of LT4, but also underscore the major unawareness regarding the separation of interfering drugs. These insights highlight the necessity of decent patient education and the importance of adequate collaboration between physicians and pharmacists to prevent LT4 malabsorption caused by food and drug interactions.

Our study revealed that nearly 25% of our study population regularly omitted breakfast to adhere to fasting LT4 ingestion. This is significantly higher than the 5% reported within the general Dutch population [13]. Because omitting breakfast has been associated with several risk factors for cardiovascular disease (including hyperlipidemia, obesity, diabetes, and atherosclerosis) [14, 15], fasting LT4 intake may indirectly result in adverse effects on cardiovascular health.

Although our questionnaire not specifically address adherence to LT4 therapy, approximately 15% of our study population reported forgetting LT4 because of the required waiting time until breakfast. We noticed that one third of our study population (33.8%) had TSH levels outside the reference range, which is roughly in line with the findings of previous studies with rates ranging from 8.5% to 42.7%, depending on which TSH levels were used as a cutoff [4-7]. These findings underline the negative impact of fasting LT4 ingestion on patient adherence and confirm considerable rates of inadequate LT4 treatment.

Many patients in our study population felt burdened with the waiting time until breakfast and would prefer nonfasting LT4 ingestion, even those with TSH levels within the reference range, with favorable QoL scores, and/or receiving LT4 treatment for many years. In a Brazilian crossover study in which 42 patients alternated between fasting and nonfasting LT4 intake, 33.2% reported a clear preference for the latter [16]. Similarly, previous studies among patients using liquid LT4 together with breakfast found that 26% to 78% preferred a nonfasting regimen [17, 18]. Our study confirms this clear patient preference for nonfasting LT4 ingestion, irrespective of the efficacy or duration of treatment.

Our findings raise the question whether the benefit of fasting ingestion outweigh its burden. We showed that fasting LT4 intake imposes a significant burden and leads to nonadherence, irregular administration of LT4, and omitting breakfast with potential adverse effects on cardiovascular health. As such, the possibility of taking LT4 with food, thereby adapting to patients' lifestyle and preferences, could potentially improve patients' adherence, satisfaction, and QoL, and may lead to more healthy breakfast habits. Despite these potential benefits, LT4 co-ingestion with breakfast is associated with a rise in TSH levels [16, 19], which necessitates more research to further explore nonfasting LT4 ingestion strategies. Bedtime ingestion (≥3 hours after dinner) has been proposed as an acceptable alternative when morning ingestion is not feasible [1, 20], but several factors make this regimen less favorable: (1) intake of LT4 60 minutes before breakfast yields more consistent TSH values [1], (2) patients might find it challenging to abstain from food several hours before bedtime, and (3) morning ingestion was traditionally established because of the circadian rhythm of TSH [3]. The development of newer LT4 formulations in liquid and soft gel form offer promising solutions by mitigating the impact of gastric pH on LT4 absorption, suggesting the possibility of co-ingestion with food, coffee, and interfering drugs [21-24]. Unfortunately, costs and reimbursement issues limit their large-scale use in many countries [25, 26]. In this study, we found that patients who took LT4 in a nonfasting state were more likely to have a TSH within the reference range, which could possibly be explained by LT4 dose adjustments in the past when TSH values were higher. This may suggest that the rise in TSH levels seen after LT4 co-ingestion with food could be prevented by increasing the LT4 dose. We are currently exploring this latter regimen in a randomized controlled trial because it might be a suitable strategy to avoid the waiting time until breakfast.

Our study has a few limitations. First, selection bias could have occurred because patients who were dissatisfied with their LT4 treatment might have been more inclined to participate, potentially leading to an overestimation of the burden associated with postponing breakfast. However, given the high response rate (88.6%) of our survey, we expect this effect to be small. Second, our study could potentially underestimate the rate of received instructions on proper LT4 ingestion by physicians and pharmacists as the majority of our study population had been on LT4 treatment for many years and these instructions could have been forgotten. Finally, our study cohort consisted of patients that received LT4 treatment in both primary and secondary care settings, whereas in the Netherlands most patients with hypothyroidism are managed in primary care. Yet, we found no differences between the patients treated in primary and secondary care with respect to received instructions on proper LT4 ingestion, patient adherence to these instructions, experienced burden with postponing breakfast, and/or preferences for nonfasting ingestion.

In conclusion, our study highlights the significant challenges and burdens associated with postponing breakfast to adhere to the current recommendation for fasting LT4 ingestion, leading to nonadherence, irregular LT4 intake, and omitting breakfast with potential adverse effects on QoL and cardiovascular health. Given the clear preferences towards LT4 ingestion together with breakfast, further research into alternative nonfasting administration methods without compromising treatment efficacy is warranted.

## Data Availability

Some or all datasets generated during and/or analyzed during the current study are not publicly available but are available from the corresponding author on reasonable request.

## Abbreviations

FT4: free thyroxine
IQR: interquartile range
LT4: levothyroxine
OR: odds ratio
QoL: quality of life
TSH: thyroid-stimulating hormone
